# A Review of Innovations in Rhegmatogenous Retinal Detachment Surgical Techniques

**DOI:** 10.1155/2017/4310643

**Published:** 2017-05-07

**Authors:** Achia Nemet, Ala Moshiri, Glenn Yiu, Anat Loewenstein, Elad Moisseiev

**Affiliations:** ^1^Department of Ophthalmology, Tel Aviv Medical Center Affiliated to the Sackler School of Medicine, Tel Aviv University, Tel Aviv, Israel; ^2^Department of Ophthalmology & Vision Science, University of California Davis Eye Center, Sacramento, CA, USA

## Abstract

Rhegmatogenous retinal detachment (RRD) requires surgical intervention for its repair. There are variable techniques used for this purpose, and they are all being continuously refined. In this review, we detail the recent innovations in surgical management of RRD and proliferative vitreoretinopathy (PVR).

## 1. Introduction

Rhegmatogenous retinal detachment (RRD) is defined as the separation of the neurosensory retina from the retinal pigment epithelium (RPE) layer due to the presence of retinal breaks. Usually, these breaks are caused by vitreous traction on the retina and allow the accumulation of fluid in the subretinal space [1]. RRD is frequently encountered by ophthalmologists of all subspecialty areas, and its repair is the quintessential procedure of vitreoretinal surgeons worldwide. The prevalence of RRD has been estimated to range from 6.3 to 17.9 per 100,000 people per year and has an overall lifetime risk of approximately 0.06% [1–3].

In the distant past, RRD was an untreatable condition ultimately resulting in irreversible vision loss. This has been dramatically transformed over the past decades, as effective treatments were developed and employed. This began with the invention and popularization of scleral buckling in 1951 by Charles Schepens, which had a high rate of success and became the treatment of choice for this condition [4]. In the 1970s, pars plana vitrectomy (PPV) was introduced by Robert Machemer and proved to be effective as well for the treatment of RRD. Later on, in 1986, pneumatic retinopexy was introduced by Hilton and Grizzard as an outpatient procedure capable of effectively treating select cases of RRD [5].

At the present, all three techniques—scleral buckling, pars plana vitrectomy, and pneumatic retinopexy—are used successfully for the treatment of RRD, with primary success rates of up to 90% [1]. PPV is currently the most common procedure used for the treatment of RRD [6, 7], although it should be noted that it is not necessarily better than scleral buckling. All of these procedures have undergone significant modifications since their original conception and have evolved along with advances in materials, instrumentation, and surgical techniques. These modifications have enabled these techniques to achieve their present potential, making them an armamentarium that allows retinal surgeons to repair almost all cases of RRD, with high rates of success. The purpose of this review is to detail the most recent innovations reported on techniques for RRD repair.

## 2. Recent Innovations

### 2.1. Pneumatic Retinopexy

Pneumatic retinopexy consists of intravitreal injection of gas followed by postoperative head positioning that places the gas bubble at the breaks, thus reducing traction and the passage of fluids into the subretinal space. This may be performed along with cryopexy before the gas injection or with laser photocoagulation around the breaks after the retina has been reattached. Not all RDs are suitable for treatment by pneumatic retinopexy, and soon after its introduction in the 1980s, it has been recommended to be used in cases with one or more retinal breaks within one-clock hour of the retinal arc in the upper two-thirds of the retina and sufficiently clear media to rule out the presence of other retinal breaks [8]. However, it has also been used successfully outside of these indications, for example, in cases with multiple breaks or break in the inferior one-third of the retina, as well as in pediatric patients [9, 10].

Not much has changed in the technique of pneumatic retinopexy since its original description. In cases where cryopexy is not performed, it may be difficult to visualize and localize the retinal breaks after the intravitreal gas injection. A recent series has reported that preoperative laser marking of the ora serrata at the meridians of the breaks made it easy to find them after pneumatic retinopexy has been performed. In a series of 10 such patients, all premarked retinal breaks were found and treated within 48 hours of the intravitreal gas injection [11]. The gas used for pneumatic retinopexy is usually C3F8 or SF6 at 100% expansile concentration, which allows for injection of a relatively small volume of gas that later expands and can cover a greater area of the retinal surface. One study reported on 77 patients who underwent pneumatic retinopexy with intravitreal injection of air, which achieved a long-term retinal reattachment rate of 80.5%, comparable to the reported rates of the conventional technique [12]. The advantage of using air is its faster rate of elimination, which allows the patients to regain good visual acuity sooner (5 days versus 2–4 weeks with the gases).

### 2.2. Scleral Buckling

Although the frequency of scleral buckling has been gradually declining since the introduction of pars plana vitrectomy, it is still a very effective technique that is still in use today. Scleral buckling originated in the 1950s and has undergone many changes in its materials, instrumentation, and surgical technique. Nevertheless, some innovations are still being reported on its use. Traditionally, identification and treatment of retinal breaks in scleral buckle surgeries were performed by indirect ophthalmoscopy. In recent years, several studies have reported on the use of fiber-optic endoillumination for this purpose, which allowed for the identification and treatment of retinal breaks to be performed with the use of a wide-field viewing apparatus. This was first described in 2008, in a series of 16 patients in whom a torpedo-style chandelier light source was used through an uncannulated sclerotomy [13]. Later refinements of this concept included the use of a fiber-optic chandelier light source through a standard transscleral cannula [14] or twin uncannulated 27-gauge chandeliers [15]. Potential advantages of this technique include improved visualization and the ability of trainees and the entire surgical team to share the intraoperative view of the surgeon [16].

Chandelier-assisted scleral buckling has also led to new techniques of subretinal fluid drainage. In one case, it has been reported to help in the direct visualization of the retina during external subretinal fluid drainage [17]. In another case, the chandelier light microcannula was used to inject balanced salt solution in order to maintain intraocular pressure and push the subretinal fluid through an external transscleral cannula under direct visualization [18].

Classic scleral buckling also typically includes a large or 360-degree peritomy. A recent study reported a technique for segmental buckling through a small conjunctival opening, which was used successfully in 46 patients with uncomplicated rhegmatogenous retinal detachment [19]. This technique includes performing a 5 to 6 mm radial conjunctival incision corresponding to the retinal break without cutting the limbal conjunctiva and Tenon's capsule, followed by cryopexy and implantation of a minimal segmental buckle that was fixed with one to two sutures through the conjunctival opening, which was later closed via layered closure. Cosmetic recovery was rapid and excellent.

An innovative technique has recently been described for suprachoroidal buckling. In this technique, an illuminated catheter is inserted into the suprachoroidal space and navigated to any desired location where peripheral breaks are present, where a long-lasting hyaluronic acid filler can be injected to create internal choroidal indentation. This can be performed without or in combination with vitrectomy and has been used successfully for the treatment of patients with retinal detachment [20, 21].

### 2.3. Pars Plana Vitrectomy

As mentioned previously, PPV is currently the most commonly used procedure for the repair of RRD [6, 7]. Over recent decades, this surgical procedure has been progressively improved due to technological advances, such as the development of small-gauge instrumentation and the use of intraocular perfluorocarbon liquid, silicone oil, and gases. All of these have played a part in making PPV a highly effective technique for repair of simple and complex RRDs, and new modifications are still being made.

The majority of PPVs performed today are small-gauge (23–27 gauge), allowing for transconjuctival and often sutureless sclerotomies. Leakage from sclerotomies and hypotony is undesirable and was more of a concern when 20-gauge instrumentation was commonly used. 20-gauge transconjunctival PPVs are still performed today, although much less frequently than a decade ago. A recent study reported that hydration of the sclerotomies achieved low rates of hypotony and complications and good final visual outcome [22].

A new technique has recently been suggested for the treatment of macular retinal detachment due to macular holes in highly myopic eyes. Due to the unique anatomy of these eyes, such macular holes are relatively difficult to close. It has been suggested that using the inverted internal limiting membrane (ILM) flap technique may be beneficial in these cases. This technique has previously been reported to improve the closure rates of large and persistent macular holes [23]. In a report of 3 patients with high myopia and macular retinal detachment due to macular holes, the ILM was peeled to the rim of the macular hole and then inverted into it, and following retinal reattachment, intraocular gas or silicone oil was used for tamponade, with retinal reattachment and macular hole closure achieved in all 3 cases [24]. This technique was later compared with standard ILM peeling without flap inversion, in a retrospective study which included 22 eyes. Higher rates of macular hole closure and retinal reattachment, as well as a small but significant improvement in the final visual acuity, were achieved with this technique [25]. It has been suggested that the inverted ILM flap stimulates the proliferation of glial cells that aid in closing the hole, and this may be a useful technique for the treatment of these challenging cases. A recent comparative study has also investigated the effectiveness of combining a macular buckle with PPV and ILM peeling in eyes with extreme myopia and RD with macular hole. The group of patients who underwent combined surgery with macular buckle had a higher rate of retinal reattachment and macular hole closure than those who did not [26].

Another interesting new technique has been reported for the treatment of macular folds, which can complicate RRD surgery and have significant implication on the visual prognosis. It has been suggested that induced detachment of the macula be performed by the subretinal injection of balanced salt solution, as well as the addition of filtered air. Under these conditions, the action of gravity of the perfluorocarbon liquid in the vitreous cavity combined with an active globe manipulation (i.e., positioning the eye so the perfluorocarbon fluid moves over the area of the detached retina that is to be flattened) has been reported to achieve successful flattening of the macula in 3 patients [27].

One of the most exciting areas of active research is the development of improved retinopexy methods which could produce immediate chorioretinal adhesion of sufficient strength to obviate the need for long-term tamponade and patient positioning. Recent studies have evaluated the potential of high-frequency electric welding (HFEW) for this purpose in a rabbit model of retinal tear. One study reported that the HFEW technique was able to create an immediate retinopexy equal in strength to mature laser retinopexy, which takes about two weeks to achieve maximum adhesion [28]. Previously reported methods to achieve this same purpose include the development of biocompatible glues, analogous to fibrin, for intraoperative use at retinal breaks [29, 30]. The elimination of long-term gas tamponade and elimination of the need for patient positioning may be the next major advance in retinal detachment surgery.

### 2.4. Management of Proliferative Vitreoretinopathy

Proliferative vitreoretinopathy (PVR) complicates 5–10% of RRD cases and is the main cause of surgical failure [31]. Risk factors for the occurrence of PVR include longer RRD duration, greater extent of the detachment, associated vitreous hemorrhage, presence of intraocular inflammation, and increased retinal tear size, as well as extensive cryopexy and laser retinopexy, failure to close retinal breaks, perioperative scleral perforation, and perioperative vitreous hemorrhage [32]. Surgical management of retinal detachment complicated by PVR is often challenging.

A recent study reported on 36 eyes with active PVR causing retinal detachment and vitreous hemorrhage, which were randomized into two groups—one group received intravitreal conbercept a week prior to surgery and the other was a control group. Administration of conbercept, a recombinant fusion protein with antivascular endothelial growth factor (VEGF) activity, was found to reduce the rate of intraoperative bleeding, which can facilitate the management of these difficult cases [33].

An interesting technique has been suggested to improve retinal flattening and prevent passage of perfluorocarbon liquid into the subretinal space. After performing vitrectomy, ophthalmic viscoelastic devices (OVDs) were injected over areas where confluent retinal folds were formed with possible retinal breaks. This protective layer still allowed the perfluorocarbon liquid placed over it to achieve retinal flattening and prevented it from entering the subretinal space. This innovation has been named “the soft-shell technique” and has been reported to have been used successfully in a series of 5 patients [34].

In recent years, partially fluorinated alkanes (FALKs) were introduced as long-term heavy tamponades, which are heavier than water (in contrast to intraocular gases and silicone oil) and may be of benefit especially in the treatment of inferior RRD or PVR. One of these is F6H8, which is not routinely used due to its early dispersion and emulsification with consequent inflammatory response. A recent study investigated its use in combination with silicone oil, in a series of 22 eyes with inferior RRD with PVR, where F6H8 was used to flatten the retina and was later partially mixed with silicone oil for long-term tamponade. This combination resulted in a clear tamponade allowing postoperative visualization of the retina, with no emulsification, inflammation, or other complications. Several different combinations of F6H8/SO were used in this study—30/70, 40/60, 50/50, 60/40, and 70/30. The best results were reported with F6H8/SO ratios between 50/50 and 30/70 [35].

Surgical management of PVR may require extensive peeling of membranes. Although these are not the tractional membranes encountered in the eyes with diabetic tractional retinal detachment, they may be similarly difficult to peel. Two recent publications have described a four-port approach for bimanual dissection of membranes in patients with diabetic tractional retinal detachment [36, 37]. The suggested technique includes an infusion port, 2 ports used by the surgeon for bimanual manipulation, and a fourth port through which the assistant holds and controls the light source. Although the use of a chandelier light source can also allow for bimanual manipulation of the retina, in this technique, the light source can still be controlled and directed closely at the area of interest. It is possible that this technique may be of use in the management of cases with severe PVR as well.

Another option is planning a staged surgery—an initial surgery to repair the retinal detachment in which tamponade is achieved with perfluorocarbon liquid and left for 2 to 3 weeks, followed by a second procedure in which it is removed. A recent study reported good results with this technique in 44 eyes with retinal detachment complicated by grade C PVR [38].

## 3. Conclusion

The surgical treatment of RRD has come a long way over the past decades, making the once untreatable condition a very manageable one. Significant advances have been made, and a variety of techniques are now available, with new instruments and modifications constantly being reported. In this review, we focused on the most recent innovations in the treatment of RRD and PVR; however, as progress continues, further improvements are expected in the future.
